# Supplementary material for: Harnessing the power of eDNA metabarcoding for the detection of deep-sea fishes
Source: PLoS One. 2020 Nov 4;15(11):e0236540. doi: 10.1371/journal.pone.0236540 (PMC7641347; doi:10.1371/journal.pone.0236540)
Supplement: S2 Fig — The blue line represents the predicted values based on a generalized linear model with 95% confidence intervals shown in gray. (PDF) [file pone.0236540.s002.pdf]

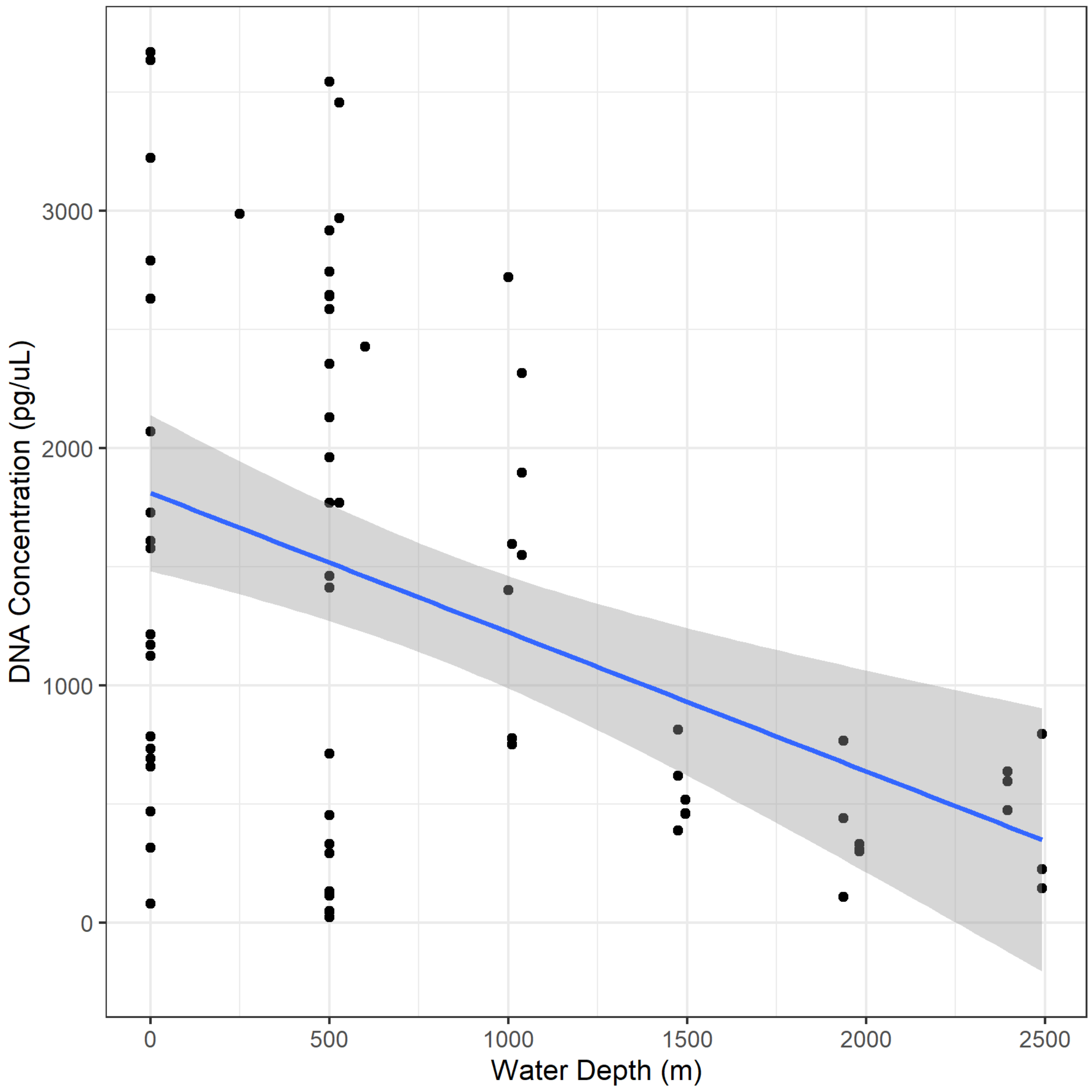

S2 Fig. Scatterplot plot comparing water sampling depth and DNA concentration for eDNA water samples collected in the Labrador Sea in 2019. The blue line represents the predicted values based on a generalized linear model with 95% confidence intervals shown in gray.
